# The symptom of vaginal bulging in nulliparous women aged 25–64 years: a national cohort study

**DOI:** 10.1007/s00192-018-3684-5

**Published:** 2018-06-23

**Authors:** Maria Gyhagen, Jwan Al-Mukhtar Othman, Sigvard Åkervall, Ida Nilsson, Ian Milsom

**Affiliations:** 1 Gothenburg Continence Research Centre, Institute of Clinical Sciences, Sahlgrenska Academy at Gothenburg University, Gothenburg, Sweden; 2 Department of Obstetrics and Gynecology, Södra Älvsborgs Hospital, 501 82 Borås, Sweden; 3 Department of Obstetrics and Gynecology, Sahlgrenska Academy at Gothenburg University, Gothenburg, Sweden; 4 Institute of Clinical Sciences, Sahlgrenska Academy at Gothenburg University, Gothenburg, Sweden

**Keywords:** Epidemiology, Nullipara, Spectrum bias, Symptomatic pelvic organ prolapse

## Abstract

**Introduction and hypothesis:**

Vaginal bulging is considered the key symptom for genital organ prolapse. The aim was to investigate the age-related prevalence and frequency of symptomatic pelvic organ prolapse (sPOP) and other pelvic floor symptoms in nonpregnant nullipara aged 25–64 years.

**Methods:**

This national postal and web-based questionnaire survey was conducted in 2014 and included four independent random samples of women aged 25–34, 35–44, 45–54, and 55–64 years. The association of sPOP with demographics and with other pelvic floor conditions and with clustering to other pelvic floor conditions, was presented in women with and without sPOP. Logistic regression was used to identify and rank variables associated with symptomatic prolapse.

**Results:**

The response rate was 52% (*n* = 10,187) and 726 nullipara confirmed sPOP. Women with sPOP were younger (*p* < 0.001), shorter (*p* < 0.001), and more often overweight and obese (*p* < 0.01) compared with asymptomatic women. Previous surgery for prolapse was reported by 15 women only (0.16%). Symptomatic POP decreased from 9.8% in the youngest age group (25–34 years) to 6.1% in the oldest (55–64 years) (*p* < 0.0001). Symptomatic POP was more often experienced as bothersome (*p* = 0.012), and aggravated by straining and heavy lifting (*p* = 0.003), in older women. Vaginal/vulval chafing/rubbing feeling was most prevalent among the youngest 14.2%, decreasing to 7.8% among the oldest (<0.0001). This symptom occurred three to five times more often in those with sPOP (*p* < 0.0001). Clustering of pelvic floor symptoms was four times more prevalent in women with sPOP (23.2% versus 6.1%) (*p* < 0.0001).

**Conclusions:**

The high prevalence of sPOP in this study was contradictory to most earlier reports, which have shown that genital prolapse is rare in nullipara. The explanation of our results may be the low probability of the clinical condition, the dominance of weak and infrequent symptoms, and not least clustering of alternative conditions mimicking sPOP.

**Electronic supplementary material:**

The online version of this article (10.1007/s00192-018-3684-5) contains supplementary material, which is available to authorized users

## Introduction

Childbirth is the dominant etiological factor for the development of pelvic organ prolapse (POP) [1, 2], resulting in a huge health care burden [3]. However, rare cases of POP, linked to other factors, have been reported in nulliparous women [4–7]. The overall rarity of POP in nullipara is confirmed by the low proportion of POP surgery in this group (0.55–1.5%) [5, 8], which may be explained by its absence or the presence of low pelvic organ prolapse quantification (POPQ) stages (0, 1, and 2) on clinical examination up to 60 years of age [9]. A number of earlier studies have shown that clinical POP with symptoms in nulliparous women <60 years occurs in less than 2–3% [9–11].

However, clinical examination is not considered feasible for large studies because of its workload, costs, and potential sampling bias [12]. Screening based on symptom questionnaires therefore predominates. In a survey of US women the prevalence of symptomatic POP (sPOP) in the nulliparous subgroup (*n* = 396) was 0.6% [13]. In questionnaire studies on samples of nulliparous women from Ireland (*n* = 1,484) and Sweden (*n* = 656), the prevalence sPOP was 1.1–2.4% [14, 15]. The reliability of screening methods for POP based on symptoms in low-risk populations has been questioned because of potential spectrum bias [12]. This concern is particularly relevant for nulliparous women, as they are often used as healthy controls to estimate the effects of childbirth on the integrity of the pelvic floor.

Tegerstedt et al. have validated the reliability of the question “Do you have a sensation of tissue protrusion (vaginal bulge) from your vagina?” to identify genital organ prolapse in a randomly selected population of women aged 30–79 years with mixed parity [15]. The primary aim of this study was to utilize this question to identify sPOP in a national cohort of nulliparous women aged 25–64 years of age. A secondary analysis was performed to study the association between “vaginal bulging” and other pelvic floor symptoms and disorders to identify possible sources of spectrum bias.

## Materials and methods

Ethical approval for the study was obtained from the Regional Ethical Review Board (reference no. 776-13; 18 November 2013). All women gave their written consent before participation.

This national postal and web-based questionnaire survey was conducted in 2014. The potential study population was identified by Statistics Sweden from the Total Population Register (TPR) and comprised women who had not given birth and who were 25–64 years of age. The TPR includes data on name, place of residence, gender, age, civil status, place of birth, citizenship, immigration, biological and adopted children, etc., and is updated every 6th week. Out of 625,810 eligible women, 20,000 were invited to participate. The participants comprised four, independent, random samples, stratified by decades of age (25–34 to 55–64 years). An introductory letter about the study, which included login credentials to a web-form, was sent to all women and was followed by a postal questionnaire. After three mailing cycles during a 4-month period, 10,187 women had responded. The web-form was used by 52% of the responders. Questionnaire data showed that 194 women were pregnant, 525 were parous, 7 lacked information about parity, and 264 declined participation or returned an unusable form. These 990 women were excluded. The final study population comprised 9,197 women. Misdiagnosis of parity (*n* = 525) was related to immigration in 337 women.

Three separate validated questionnaires created by Tegerstedt et al. [16], Sandvik et al. [17], and Jorge and Wexner [18] were combined into one. The questionnaire is attached as Appendix 1. The questionnaire included items about height and weight, a control question regarding ongoing pregnancy and births, menstrual status, hysterectomy, menopausal status and hormone treatment, major pelvic floor disorders, etc. Missing answers were lowest for “taking medication for UI” (0.3%) and highest for “sought doctor for UI” (1.9%). Symptomatic POP was defined by the question “Do you have a sensation of tissue protrusion (a vaginal bulge) from your vagina? “ with the alternative answers Never = No, Infrequently/Sometimes/Often = Yes [16]. The question has previously been validated in women with mixed parity [15, 16]. Fecal incontinence (FI) was defined by affirming involuntary loss of liquid or solid feces (Infrequently/Sometimes/Often) [18]. In women with sPOP, bothersome symptoms were defined by the question “How do these vaginal symptoms affect you?” Not bothersome = No problem/A small nuisance and Bothersome = Some bother/Much bother/A major problem. Women were also questioned regarding surgery for prolapse. Heredity was assessed by the question “Has your mother suffered from prolapse?” (Yes/No/Do not know). Those who answered, “Do not know” were excluded from the analysis of heredity.

Urinary incontinence (UI), subtypes of UI, urgency/overactive bladder (OAB), and nocturia were defined according to the International Urogynecological Association (IUGA)/International Continence Society (ICS) definition [19]. UI was defined by the question “Do you have involuntary loss of urine.” Stress urinary incontinence was defined as involuntary loss of urine in connection with coughing, sneezing, laughing, or lifting heavy items. Urge urinary incontinence was present if loss of urine was in connection with a sudden and strong urge to void, and mixed urinary incontinence if both components were present. Urgency/OAB was defined as “Do you have urinary urgency with a sudden and strong urge to void which is hard to postpone?” Body mass index (BMI = kg/m^2^) was calculated from the weight and height given in the questionnaire.

Statistical analyses were performed using SPSS software (SPSS Statistics 22; IBM Corp, Armonk, NY, USA). Chi-squared test was used to compare categorical and Student’s* t* test to compare continuous variables. Crude and adjusted prevalence and proportion and 95% confidence intervals (CI) for all outcomes were calculated for each 10-year category. Adjusted prevalence and odds ratio (OR) were calculated from the logistic regression model, taking age and BMI into account. Age- and BMI-dependent differences for various aspects of vulvar/vaginal symptoms were analyzed, with the youngest group (25–34 years) as reference. A *p* value of <0.05 was considered statistically significant. A logistic regression model was used to assess predictors for sPOP presented as OR and 95% confidence interval (95%CI). Potential predictors used in the analysis were: age < 35 years, BMI ≥ 30 kg/m^2^, childhood nocturnal enuresis, chafing, OAB, FI, and UI.

## Results

The response rate was 52.2% (*n* = 10,187), increasing from 44.7% among the youngest (25–34 years), to 62.4% among the oldest women (55–64 years). The number of eligible women was 9,923 of which 9,136 women answered the question about sPOP. Women with sPOP (*n* = 726) were younger, shorter, more often overweight and obese, compared with those without sPOP (*n* = 8,410; Table 1).

**Table 1:** Characteristics of the nulliparous women with and without symptomatic pelvic organ prolapse (*n* = 9,136)

Cohort characteristics	sPOP *n* = 726	Non-sPOP *n* = 8,410	*p* value
	Mean (%) (95%CI)	Mean (%) (95%CI)	
Age, years	39.0 (38.2–39.9)	40.9 (40.7–41.2)	<0.001
Age ≥ 45 years^a^	29.1 (25.8–32.4)	35.9 (34.9–36.9)	<0.001
Weight, kg	69.7 (68.5–70.9)	69.2 (68.9–69.5)	= 0.41
Height, cm	165.7 (165.2–166.3)	167.0 (166.8–167.1)	<0.001
BMI, kg/m^2^	25.4 (25.0–25.8)	24.8 (24.7–24.9)	<0.01
BMI ≥30, kg/m^2^^b^	17.6 (14.8–20.4)	13.8 (13.0–14.5)	<0.01
Hysterectomy	4.5 (3.0–6.1)	3.8 (3.4–4.2)	= 0.33
Postmenopausal	13.8 (11.3–16.3)	17.5 (16.7–18.3)	= 0.011
Estrogen treatment	1.9 (0.9–2.9)	2.0 (1.7–2.3)	= 0.90
Childhood nocturnal enuresis	16.7 (15.7–17.7)	10.9 (10.2–11.6)	<0.001
Family history of sPOP^c^	22.4 (17.5–27.3)	13.3 (12.3–14.3)	<0.001

The proportion of missing data varied between 0.7% for childhood nocturnal enuresis (lowest) to 2.8% for family history (highest)

*BMI* body mass index, *sPOP* symptomatic pelvic organ prolapse

^a^Proportion (%) of women ≥45 years

^b^Proportion (%) of women with BMI ≥30, kg/m^2^

^c^Family history of sPOP, 4,310/9,136 women answered “Do not know” and were not included in the analysis (48.5%)

The prevalence of having a “bulging” sensation “Infrequently, or more often” was 7.9%, “Sometimes, or more often” 2.9%, and “Often” 0.4% in the total cohort (not shown in Table 2). The BMI-adjusted prevalence of sPOP decreased across ages from 9.8% among the youngest to 6.1% in the oldest group (Table 2). Fifteen of the nulliparous women (0.16%) had undergone surgery for prolapse (not shown in Table 2). Bothersome sPOP was more common in the oldest age group, 21.7% (Table 2). Bulging sensation reported as “Often” was less common, increasing with age from 3.9 to 7.0%. Reporting aggravation of bulging during heavy lifting was twice as common among the oldest women compared with the youngest women (22.5% versus 10.9% Table 2). The symptom “Vaginal/vulval chafing/rubbing feeling” was most prevalent among the youngest 14.2%, decreasing across the ages to 7.8% among the oldest women (Table 2).

**Table 2:** Prevalence and severity of pelvic floor symptoms according to age

	25–34 years *n* = 3,316	35–44 years *n* = 2,589	45–54 years *n* = 1,536	55–64 years *n* = 1,695	*p* value for trend
	Crude/adjusted^a^ % (95% CI)	Crude/adjusted^a^ % (95% CI)	Crude/adjusted^a^ % (95% CI)	Crude/adjusted^a^ % (95% CI)	
Prevalence of sPOP parameters in all nulliparous women (*n* = 9,136), missing = 61					
Infrequent or more often *n* = 726	9.6 (8.6–10.6) 9.8 (8.8–10.9)	7.7 (6.6–8.7) 7.4 (6.5–8.5)	6.7 (5.5–8.0) 6.3 (5.2–7.7)	6.4 (5.2–7.5) 6.1 (5.1–7.4)	<0.0001
Sometimes or more often *n* = 263	3.5 (2.9–4.2) 3.6 (3.0–4.4)	2.7 (2.2–3.4) 2.6 (2.2–3.3)	2.2 (1.6–3.1) 2.1 (1.4–3.0)	2.5 (1.8–3.3) 2.3 (1.8–3.2)	=0.013
Often *n* = 35	0.4 (0.2–0.6) 0.4 (0.2–0.7)	0.3 (0.1–0.5) 0.3 (0.1–0.6)	0.5 (0.1–0.8) 0.3 (0.1–0.8)	0.5 (0.1–0.8) 0.4 (0.2–0.9)	=0.463
Chafing/rubbing feeling *n* = 946	13.9 (12.7–15.1) 14.2 (13.0–15.5)	8.6 (7.5–9.6) 8.5 (7.4–9.6)	8.2 (6.7–9.5) 7.7 (6.4–9.1)	8.1 (6.8–9.4) 7.8 (6.6–9.2)	<0.0001
Proportion of sPOP parameters in women with sPOP (*n* = 726)					
Infrequent *n* = 463	63.4 (58.1–68.7) 63.1 (57.5–68.3)	64.1 (57.5–70.8) 65.8 (58.8–72.2)	67.0 (57.9–76.1) 69.0 (59.3–77.3)	61.1 (51.9–70.3) 63.0 (53.2–71.7)	0.474*
Sometimes *n* = 228	32.8 (27.6–38.0) 32.9 (27.9–38.4)	31.8 (25.3–38.3) 30.3 (24.2–37.2)	26.2 (17.7–34.7) 25.3 (17.7–34.7	31.5 (22.7–40.2) 29.9 (21.9–39.4)	
Often *n* = 35	3.8 (1.7–5.9) 3.9 (2.2–6.8)	4.0 (1.3–6.8) 4.0 (2.0–7.8)	6.8 (1.9–11.7) 5.8 (2.6–12.4)	7.4 (2.5–12.3) 7.0 (3.5–13.7)	
Bothersome	12.2 (8.1–16.4) 12.1 (8.5–17.0)	14.5 (8.8–20.2) 13.7 (9.0–20.4)	22.7 (13.2–32.1) 20.9 (13.1–31.7)	21.9 (12.4–31.4) 21.7 (13.6–32.8)	=0.012
Aggravation during straining and heavy lifting	10.8 (6.8–14.8) 10.9 (7.4–15.7)	18.7 (12.2–25.2) 17.7 (12.2–26.1)	18.9 (10.0–27.8) 17.5 (10.4–27.8)	24.7 (14.8–34.5) 22.5 (14.1–33.7)	=0.003

*CI* confidence interval

*p for trend

^a^Adjusted for body mass index

**Fig. 1:**
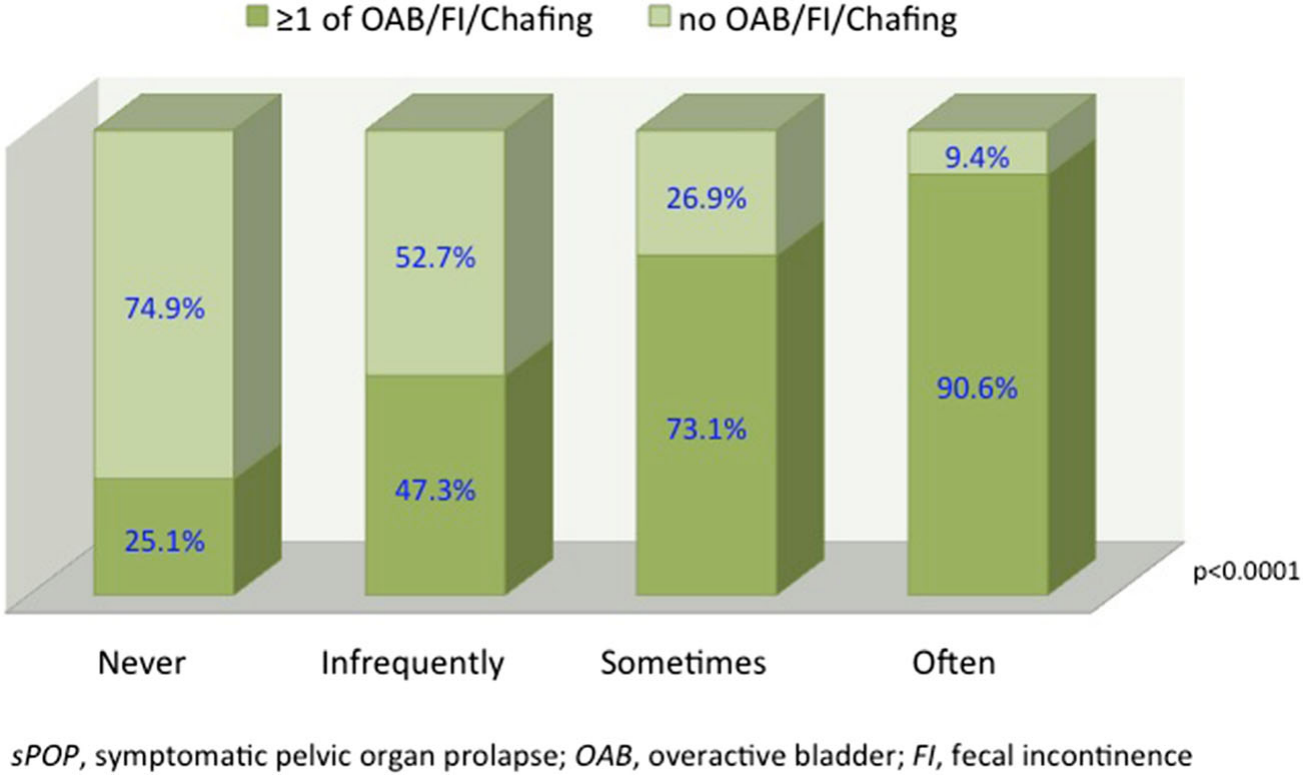
Co-occurring overactive bladder (*OAB*)/fecal incontinence (*FI*)/chafing symptoms grouped according to symptomatic pelvic organ prolapse (*sPOP*) symptom frequency

**Fig. 2:**
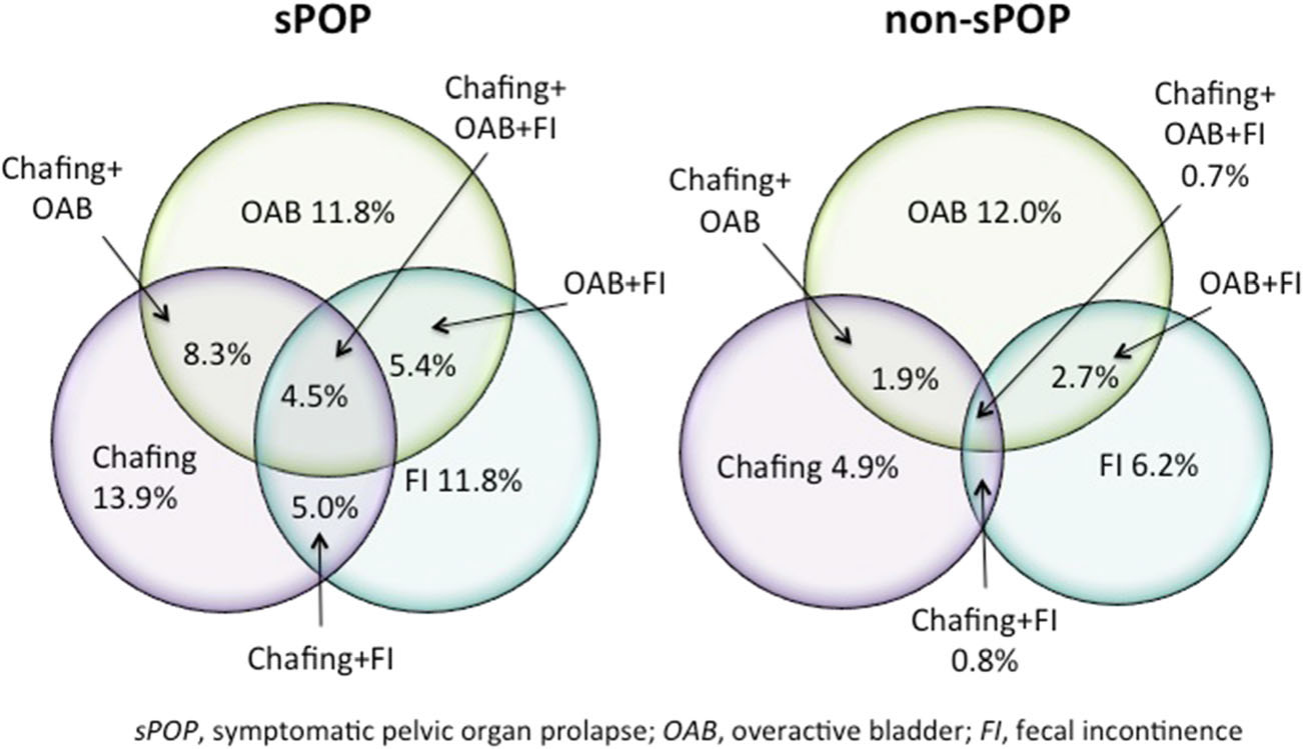
Co-occurring symptoms in women with and without sPOP

The association between “chafing” and sPOP was strong in all age groups (OR 4.29–6.87) and occurred three times more often (e.g., 36.4% versus 11.8%) in the youngest age group (Table 3). Urgency/OAB prevalence was approximately doubled in women with sPOP. FI occurred almost three times more often across ages in those with sPOP (22.9% in women 25–34 years) compared with those without sPOP (8.5%). In contrast, the association between sPOP and UI and all subtypes of UI was inconsistent and weaker across ages (Table 3). Having one or more concomitant genital symptoms, chafing, OAB or FI was associated with an increasing frequency of a bulging sensation (Fig. 1). Clustering of pelvic floor symptoms was four times more prevalent in women with sPOP (23.2% versus 6.1%; Fig. 2).

**Table 3:** Crude and adjusted prevalence of pelvic floor symptoms and disorders in women with or without symptomatic pelvic organ prolapse according to age (*n* = 9,136)

		Age 25–34 Crude/adjusted % (95%CI) *n* = 3,316	Adjusted^a^ OR (95% CI) *p* value	Age 35–44 Crude/adjusted % (95%C) *n* = 2,589	Adjusted^a^ OR (95% CI) *p* value	Age 45–54 Crude/adjusted % (95%CI) *n* = 1,536	Adjusted^a^ OR (95% CI) *p* value	Age 55–64 Crude/adjusted % (95%CI) *n* = 1,695	Adjusted^a^ OR (95% CI) *p* value	*p* value for trend
Chafing/rubbing feeling *n* = 940	sPOP	36.7 (31.4–42.0) 36.4 (31.2–42.0) *n* = 116	4.29 (3.31–5.55) <0.0001	32.1 (25.6–38.7) 32.4 (26.2–39.4) *n* = 63	6.85 (4.86–9.65) <0.0001	25.2 (16.9–33.6) 24.6 (17.1–33.9) *n* = 26	4.67 (2.83–7.72) <0.0001	27.6 (19.1–36.2) 26.7 (19.0–36.0) *n* = 29	5.24 (3.24–8.46) <0.0001	<0.0001
	Non-sPOP	11.5 (10.3–12.6) 11.8 (10.6–13.0) *n* = 344		6.6 (5.6–7.6) 6.5 (5.6–7.6) *n* = 157		6.9 (5.6–8.2) 6.5 (5.3–7.9) *n* = 98		6.8 (5.5–8.0) 6.6 (5.5–7.9) *n* = 107		
OAB *n* = 1,810	sPOP	26.8 (22.1–31.9) 27.3 (22.6–32.8) *n* = 85	2.29 (1.74–3.01) <0.0001	31.4 (26.0–39.3) 31.2 (25.0–38.2) *n* = 60	2.32 (1.67–3.22) <0.0001	42.4 (33.4–53.2) 41.9 (32.4–52.1) *n* = 42	2.39 (1.56–3.66) <0.0001	46.2 (38.6–57.6) 45.5 (36.2–55.2) *n* = 49	1.90 (1.27–2.85) <0.002	<0.0001
	Non-sPOP	13.6 (12.4–14.9) 14.2 (12.9–15.5) *n* = 406		15.7 (14.4–17.4) 15.7 (14.3–17.3) *n* = 373		23.5 (21.4–25.9) 22.8 (20.7–25.1) *n* = 333		29.5 (27.6–32.2) 29.0 (26.8–31.4) *n* = 462		
UI *n* = 1,477	sPOP	19.1 (14.7–23.4) 19.1 (15.0–23.9) *n* = 60	1.83 (1.35–2.50) <0.0001	22.8 (17.0–28.7) 21.1 (15.9–27.3) *n* = 45	1.76 (1.23–2.53) =0.002	38.0 (28.5–47.5) 35.1 (26.2–45.1) *n* = 38	2.40 (1.55–3.73) <0.0001	31.8 (23.0–40.6) 29.9 (21.9–39.2) *n* = 34	1.49 (0.97–2.30) =0.071	<0.0001
	Non-sPOP	10.7 (9.6–11.8) 11.5 (10.3–12.7) *n* = 321		13.7 (12.3–15.1) 13.1 (11.8–14.5) *n* = 326		20.0 (17.9–22.1) 18.1 (16.2–20.2) *n* = 285		23.5 (21.4–25.6) 21.8 (19.8–24.0) *n* = 368		
SUI *n* = 585	sPOP	7.3 (4.5–10.3) 7.2 (4.8–10.7) *n* = 23	1.44 (0.90–2.29) =0.129	11.1 (6.8–15.6) 10.2 (6.7–15.2) *n* = 22	1.89 (1.16–3.09) <0.010	10.7 (4.9–17.3) 8.6 (4.5–15.8) *n* = 11	1.06 (0.52–2.17) =0.87	12.0 (6.1–18.7) 11.3 (6.6–18.7) *n* = 13	1.75 (0.95–3.23) =0.075	=0.004
	Non-sPOP	4.8 (4.0–5.6) 5.1 (4.4–6.0) *n* = 143		5.8 (4.9–6.8) 5.6 (4.7–6.5) *n* = 139		8.4 (7.0–9.9) 7.7 (6.5–9.2) *n* = 120		7.2 (6.0–8.6) 6.7 (5.6–8.0) *n* = 114		
UUI *n* = 259	sPOP	3.8 (1.7–6.0) 3.9 (2.2–6.7) *n* = 12	2.03 (1.08–3.85) =0.029	2.0 (0.1–4.0) 2.1 (0.8–5.4) *n* = 4	0.75 (0.27–2.09) =0.583	3.9 (0.2–7.8) 4.1 (1.5–10.4) *n* = 4	1.29 (0.46–3.68) =0.629	1.9 (−0.7–4.5) 1.9 (0.5–7.4) *n* = 2	0.40 (0.10–1.67) =0.209	=0.707
	Non-sPOP	1.8 (1.4–2.3) 1.9 (1.4–2.4) *n* = 55		2.8 (2.1–3.4) 2.8 (2.2–3.5) *n* = 66		3.1 (2.3–4.1) 3.1 (2.3–4.2) *n* = 45		4.5 (3.5–6.6) 4.1 (1.5–10.4) *n* = 71		
MUI *n* = 429	sPOP	4.7 (2.4–7.2) 4.7 (2.8–7.6) *n* = 15	2.13 (1.19–3.80) =0.011	7.1 (3.5–10.7) 5.9 (3.4–9.9) *n* = 14	2.31 (1.24–4.30) =0.008	12.6 (6.5–19.8) 11.5 (6.7–19.1) *n* = 13	2.20 (1.17–4.13) =0.014	14.8 (8.4–22.1) 13.0 (88.0–20.5) *n* = 16	1.64 (0.93–2.89) =0.088	<0.0001
	Non-sPOP	2.2 (1.7–2.7) 2.3 (1.8–2.9) *n* = 65		2.8 (2.2–3.5) 2.5 (2.0–3.2) *n* = 67		6.5 (5.3–7.8) 5.3 (4.3–6.6) *n* = 93		9.2 (7.9–10.8) 8.0 (6.8–9.5) *n* = 146		
FI *n* = 1,079	sPOP	23.3 (18.7–28.0) 22.9 (18.6–28.0) *n* = 74	3.23 (2.40–4.35) <0.0001	25.1 (19.0–31.2) 23.1 (17.7–29.6) *n* = 49	2.92 (2.03–4.19) <0.0001	38.0 (28.5–47.5) 36.8 (27.9–46.8) *n* = 38	4.05 (2.61–6.30) <0.0001	35.5 (26.4–44.6) 34.4 (25.9–44.0) *n* = 38	3.25 (2.12–5.00) <0.0001	<0.0001
	Non-sPOP	8.2 (7.2–9.2) 8.5 (7.5–9.6) *n* = 245		9.4 (8.2–10.6) 9.2 (8.1–10.5) *n* = 223		13.3 (11.5–15.1) 12.6 (11.0–14.4) *n* = 189		14.2 (12.5–16.0) 13.7 (12.0–15.5) *n* = 223		
UI + FI *n* = 364	sPOP	5.7 (3.1–8.3) 5.3 (3.3–8.5) *n* = 18	3.09 (1.76–5.44) <0.0001	6.6 (3.1–10.1) 5.2 (2.9–9.1) *n* = 13	1.99 (1.03–3.85) =0.041	19.0 (11.3–26.7) 16.5 (10.5–24.9) *n* = 19	4.30 (2.42–7.64) <0.0001	15.0 (8.2–21.7) 13.4 (8.2–20.9) *n* = 16	2.54 (1.43–4.52) =0.002	<0.0001
	Non-sPOP	1.7 (1.3–2.2) 1.8 (1.4–2.4) *n* = 52		2.8 (2.2–3.5) 2.7 (2.1–3.4) *n* = 68		5.3 (4.2–6.5) 4.5 (3.6–5.7) *n* = 76		6.5 (5.2–7.7) 5.6 (4.6–6.8) *n* = 102		

*OAB* overactive bladder, *UI* urinary incontinence, *SUI* stress urinary incontinence, *UUI* urge urinary incontinence, *MUI* mixed urinary incontinence, *FI* fecal incontinence

^a^Adjusted for body mass index (kg/m^2^)

The logistic regression showed that sPOP was associated with younger age (<35 years, OR 1.45; 95% CI 1.23–1.72), but not with obesity (BMI ≥ 30) or UI. Chafing was the strongest predictor for sPOP (OR 4.25; 95% CI 3.52–5.12), whereas OAB was a significant but weak predictor (Table 4).

**Table 4:** Multivariate logistic regression of predictors for symptomatic pelvic organ prolapse (*n* = 8,752)

	sPOP 25–64 years	*p* value
	OR (95%CI)	
Age < 35 years	1.45 (1.23–1.72)	<0.0001
BMI ≥ 30	1.24 (0.99–1.55)	0.058
Childhood nocturnal enuresis	1.32 (1.05–1.65)	0.017
Chafing/rubbing feeling	4.25 (3.52–5.12)	<0.0001
Urgency/OAB	1.47 (1.20–1.81)	0.0002
Fecal incontinence	2.58 (2.11–3.14)	<0.0001
Urinary incontinence	1.03 (0.82–1.29)	0.812

Responders and nonresponders were compared using information from the TPR, available for 99.9% of the total sample. Nonresponders were younger (56.7% of the age group 25–29 years compared with 37.3% of the age group 60–64 years), and were more likely to be immigrants (63.7%), non-Swedish citizens (67.8%), unmarried (51.2%), living in suburban or commuting municipalities (53.7%), and having a lower income and lower level of education (59.1% of those with ≤3 years of secondary education compared with 40.9% of those with >3 years secondary education and higher) [20].

## Discussion

The prevalence of sPOP in this study was high, and highest in the youngest women, decreasing with age. Conversely, surgery for prolapse was negligible in this cohort of nulliparous women aged 25–64 years of age. Nulliparous women with sPOP were shorter, more often overweight and obese, and more often reported childhood nocturnal enuresis and a family history of sPOP. The cut-off point of symptom frequency to define a positive response had a decisive influence on prevalence rates, decreasing drastically with higher frequencies. In addition, the symptom of “bulging” was strongly associated with “chafing” as a symptom and also, but somewhat weaker, with co-occurring FI and urgency/OAB. The proportion of co-occurring symptoms increased linearly with increased frequency of “bulging.”

In contrast to our results, an sPOP prevalence of 0.6% was reported in subgroups of nulliparous women from two cross-sectional studies on randomly selected US women aged ≥20 years [13]. In the survey by Nygaard et al. [13], the question for sPOP was “Do you experience bulging or something falling out you can see or feel in the vaginal area?” Two out of 396 nullipara admitted this symptom. Tegerstedt et al. [15] used a postal questionnaire to assess the prevalence of POP in a survey of 5,489 randomly selected women living in the city of Stockholm. Of 1,458 nullipara (mean age 44 years), 35 (2.4%) affirmed the question, “Do you have a sensation of tissue protrusion (vaginal bulge) from the vagina?”, which is identical to the question used in the present study [15]. In a recent questionnaire study by Cooper et al. [21] from a UK community practice of women >18 years of age (parous as well as nulliparous) two questions from the International Consultation on Incontinence Questionnaire for Vaginal Symptoms (ICIQ-VS) were used to identify POP: “Are you aware of a lump or bulge coming down in your vagina?”, and “Do you feel a lump or bulge come out of your vagina, so that you can feel it on the outside or see it on the outside?”. Interestingly, the prevalence was 8.4 and 4.9% respectively [21]. These results indicate that the content, wording, and the definition of a positive response may significantly affect the prevalence rate.

In contrast to studies based on self-reporting, the prevalence of prolapse based on clinical investigations has produced more consistent results. In a few studies, mainly of convenient samples of women seeking care at outpatient clinics, the distribution of POPQ stages in nulliparous women of different ages has been described. In four studies on a total of 607 nullipara, 3 women aged <60 years had POPQ stage ≥3 (0.5%) and 20–25% had a stage 2 prolapse [9–11, 22]. Given a selection bias due to a health deficit that adheres to outpatient samples, these results are presumably overestimates.

Case reports on rare causes of prolapse have also been reported in nulliparous women. They form a heterogeneous group with diverse pathogenic factors, such as connective tissue disorders, previous pelvic surgery, previous pelvic fractures, spinal cord injury, prolonged severe constipation, and excessive strenuous physical activity [4–7]. We had no information in the questionnaire about these conditions.

The rarity of prolapse in nullipara is supported by register data regarding prolapse surgery. According to the Swedish GynOp Register [8], 278 (0.8%) of all reconstructive prolapse procedures during the 7-year period 2010–2016 (*n* = 33,124) were performed on women who claimed to be nulliparous. The study of Lo et al. reported on 1,275 surgically managed women with a prolapse of POPQ stage ≥3. In this large sample from one Taiwanese center, collected between 2005 and 2015, 8 women were nulliparous (0.55%) [5].

Although the relationship between clinical stage and the symptoms of POP is unpredictable [23], it has been shown that symptoms increase markedly once the leading edge reaches 1 cm distal to the introitus; hence, including some patients with POPQ stage 2 and all stage ≥3 [24]. Tan et al. found that women with POPQ stage 2 had symptoms in 24% at 1 cm above introitus and in 49% at the hymenal remnant [25]. Considering the predominance of stage 0 and 1 (>80%) in nulliparous women [9–11, 22], and a distribution that is most probably skewed toward more proximal and asymptomatic stage 0–2, the prevalence of symptoms from an anatomical prolapse should theoretically be expected to be, at most, 1–2% in nulliparous women <60 years of age.

The relationship between POPQ stage 0 and the “bulging” symptom has been studied by Tegerstedt et al. [15, 16]. In 199 women, they found that “bulging” was reported to occur “Infrequently” in 6.0%, “Sometimes” in 2.5%, and “Often” in 0%. This study is a unique natural experiment and the first to report the proportion of indisputably false-positive responses in a large group of women who did not have a clinically detectable prolapse. Further, their results are very similar to the result of the present study of 5.1, 2.4, and 0.4% (Infrequent/Sometimes/Often). This conformity is of significance as both Tegerstedt et al. and the present study used the same question for sPOP, analyzed a randomly selected Swedish population, and were conducted within a 10-year period. It therefore seems reasonable to expect that both nulliparous women <65 years of age and women without an anatomical prolapse self-report “bulging” (Infrequently/Sometimes/Often) in ≈8%. These data also show that the choice of cut-off point for symptom frequency to define a positive response is decisive for prevalence rates.

In this study, the prevalence and age-related trend of sPOP were counterintuitive, therefore suggesting a spectrum effect [26]. Unlike UI and FI, genital prolapse is not a uniform, easily definable pathological state, but rather a continuum ranging from barely existing to clearly present. Any distinction between a normal variant and the disease state of POP has been considered to be arbitrary [27], both on the basis of clinical examination [28] and as defined from diagnostic questions that are symptom-based, which are considered to be particularly prone to spectrum bias. In surveys with a low prevalence of the target condition, there may be more individuals in whom the condition is less severe and atypical [26], further increasing the likelihood of a spectrum effect. Nulliparous women, with clinical stages skewed toward “Not present” and some milder forms, may interpret the “bulging” question differently compared with those with genital prolapse beyond the hymen, having experienced the condition-specific symptom often and for a long time. For instance, in the study by Tegerstedt et al. the symptom of “bulging” was experienced “Sometimes or more often” in 85.5% of women with clinically confirmed POP and in 2.5% of women with POPQ stage 0 [16]. In our study, the prevalence of sPOP, defined by the cut-off frequency including only those having the symptom “Sometimes or more often,” was similar (2.9%).

The puzzle of false-positive responses among women without clinical prolapse has not yet been satisfactorily examined. If a prolapse is not present, the experience, which is perceived as “bulging” must be due to other conditions. The question about a bulging sensation is more likely to produce false-positive responses if there are coexisting conditions in the same anatomical location that elicit sensations mimicking and misinterpreted as the classic symptom [12, 26]. Young age (<35 years), overweight and obesity, and enuresis all had a significant, but weak association with the “bulging” symptom. The strong association between UI and prolapse in the literature [29] was not observed in this study, further indicating the absence of anatomical prolapse. The three strongest predictors for sPOP were chafing, urgency/OAB, and FI. The high prevalence of “chafing” among women in the youngest age group may be explained by their higher levels of physical and sexual activity. The association between the successive increase in prevalence and clustering of these conditions and increasing frequency of sPOP indicates that they may contribute to sensations that are perceived as “bulging” and translate into false-positive responses.

The strength of the design used for this study has been presented elsewhere [20]. The obvious limitation of this study was that the participants were not clinically evaluated for staging according to the POPQ. However, this would have been highly impractical and led to another type of selection bias [12]. The response rate increased with age from 43% among the youngest to 63% among the oldest, which is frequently observed and may simply reflect that older women are more likely to be compliant and respond. Younger women may preferentially respond because of symptoms that may introduce a bias toward the sick and inflate prevalence. The validity of self-report, upon which this study is based, depends on the participants’ willingness and ability to perceive, evaluate, and report correctly, which may also change with aging. Data on nonresponders also suggests a selection bias. Nonresponders were younger, more often immigrants or non-Swedish citizens, less often married, living in suburbs or commuting municipalities, and had a lower income and level of education. This indicates a lower socioeconomic status (SES) of the nonresponders. However, the association between SES and genital prolapse is not relevant to this study, which focuses on women with a low probability of the condition. Women in this study were predominantly Caucasians, which is why results should be interpreted with caution for diverse ethnic groups.

## Conclusion

Compared with earlier studies, the prevalence of sPOP in this study of nulliparous women was 3 to 20 times higher. However, according to current knowledge, anatomical prolapse in nulliparous women causing symptoms of protrusion is, simply put, almost non-existent. The high prevalence of sPOP in this study seemed to be determined by the inclusion of women with symptoms that occur “Infrequently.” Further, the prevalence of “bulging” was related to young age and the co-occurrence of chafing/urgency/FI.

## Electronic supplementary material

ESM 1 (DOCX 108 kb)

## References

[1] Leijonhufvud Å, Lundholm C, Cnattingius S, Granath F, Andolf E, Altman D (2011). Risks of stress urinary incontinence and pelvic organ prolapse surgery in relation to mode of childbirth. Am J Obstet Gynecol.

[2] Samuelsson EC, Victor AFT, Tibblin G, Svärdsudd KF (1999). Signs of genital prolapse in a Swedish population of women 20 to 59 years of age and possible related factors. Am J Obstet Gynecol.

[3] DeLancey JO (2005). The hidden epidemic of pelvic floor dysfunction: achievable goals for improved prevention and treatment. Review. Am J Obstet Gynecol.

[4] Norton P, Baker J, Sharp H, Warenski J (1990). Genitourinary prolapse: its relationship with joint mobility. Neurourol Urodyn.

[5] Lo TS, Jaili S, Uy-Patrimonio MC, Karim NB, Ibrahim R (2017). Transvaginal management of severe pelvic organ prolapse in nulliparous women. J Obstet Gynaecol Res.

[6] Wan L, Liu X (2016). Delayed-onset advanced pelvic organ prolapse after spinal cord injury in a young, nulliparous woman. Int Urogynecol J.

[7] Dietz HP, Clarke B (2005). Prevalence of rectocele in young nulliparous women. Aust New Zeal J Obstet Gynaecol.

[8] http://www.gynop.org/english/english.htm. Accessed 7 January 2018.

[9] Quiroz LH, White DE, Juarez D, Shobeiri SA (2012). Age effects on pelvic floor symptoms in a cohort of nulliparous patients. Female Pelvic Med Reconstr Surg.

[10] Swift SE (2000). The distribution of pelvic organ support in a population of female subjects seen for routine gynecologic health care. Am J Obstet Gynecol.

[11] Sze EHM, Hobbs G (2009). Relation between vaginal birth and pelvic organ prolapse. Acta Obstet Gynecol Scand.

[12] Barber MD, Neubauer NL, Klein-Olarte V (2006). Can we screen for pelvic organ prolapse without a physical examination in epidemiologic studies?. Am J Obstet Gynecol.

[13] Nygaard I, Barber MD, Kathryn L, Burgio KL, Kenton K, Meikle S (2008). Prevalence of symptomatic pelvic floor disorders in US women. JAMA.

[14] Durnea CM, Khashan AS, Kenny LC, Tabirca SS, O’Reilly BA (2014). An insight into pelvic floor status in nulliparous women. Int Urogynecol J.

[15] Tegerstedt G, Maehle-Schmidt M, Nyrén O, Hammarström M (2005). Prevalence of symptomatic pelvic organ prolapse in a Swedish population. Int Urogynecol J.

[16] Tegerstedt G, Miedel A, Maehle-Schmidt M, Nyrén O, Hammarström M (2005). A short-form questionnaire identified genital organ prolapse. J Clin Epidemiol.

[17] Sandvik H, Hunskaar S, Seim A, Hermstad R, Vanvik A, Bratt H (1993). Validation of a severity index in female urinary incontinence and its implementation in an epidemiological survey. J Epidemiol Community Health.

[18] Jorge JM, Wexner SD (1993). Etiology and management of fecal incontinence. Dis Colon Rectum.

[19] Haylen BT, de Ridder D, Freeman RM (2010). An International Urogynecological Association (IUGA)/International Continence Society (ICS) joint report on the terminology for female pelvic floor dysfunction. Int Urogynecol J.

[20] Al-Mukhtar Othman J, Åkervall S, Milsom I, Gyhagen M (2017). Urinary incontinence in nulliparous women aged 25–64 years: a national survey. Am J Obstet Gynecol.

[21] Cooper J, Annappa M, Dracocardos D, Cooper W, Sara Muller S, Mallen C (2015). Prevalence of genital prolapse symptoms in primary care: a cross-sectional survey. Int Urogynecol J.

[22] O'Boyle AL, Woodman PJ, O'Boyle JD, Davis GD, Swift SE (2002). Pelvic organ support in nulliparous pregnant and nonpregnant women: a case control study. Am J Obstet Gynecol.

[23] Ellerkmann RM, Cundiff GW, Melick CF, Nihira MA, Leffler K, Bent AE (2001). Correlation of symptoms with location and severity of pelvic organ prolapse. Am J Obstet Gynecol.

[24] Swift SE, Tate SB, Nicholas J (2003). Correlation of symptoms with degree of pelvic organ support in a general population of women: what is pelvic organ prolapse?. Am J Obstet Gynecol.

[25] Tan JS, Lukacz ES, Menefee SA, Powell CR, Nager CW (2005). Predictive value of prolapse symptoms: a large database study. Int Urogynecol J Pelvic Floor Dysfunct.

[26] Ransohoff DF, Feinstein AR (1978). Problems of spectrum and bias in evaluating the efficacy of diagnostic tests. N Engl J Med.

[27] Weber AM, Abrams P, Brubaker L (2001). The standardization of terminology for researchers in female pelvic floor disorders. Int Urogynecol J Pelvic Floor Dysfunct.

[28] Bump RC, Mattiasson A, Bø K (1996). The standardization of terminology of female pelvic organ prolapse and pelvic floor dysfunction. Am J Obstet Gynecol.

[29] Gyhagen M, Bullarbo M, Nielsen T, Milsom I (2013). Prevalence and risk factors for pelvic organ prolapse 20 years after childbirth: a national cohort study in singleton primiparae after vaginal or caesarean delivery. BJOG.

