# Supplementary material for: The symptom of vaginal bulging in nulliparous women aged 25–64 years: a national cohort study
Source: Int Urogynecol J. 2018 Jun 23;30(4):639–47. doi: 10.1007/s00192-018-3684-5 (PMC6451022; doi:10.1007/s00192-018-3684-5)
Supplement: Supplementary file 1 — (DOCX 108 kb) [file 192_2018_3684_MOESM1_ESM.docx]

| **APPENDIX** | | |
| --- | --- | --- |
| Q1 | How tall are you? | cm |
| Q2 | How much do you weigh? | kg |
| Q3 | Have you given birth? | Yes  If so how many children have you had?­­ _______  No I have not given birth |
| Q4 | Do you still have menstrual periods? | Yes/No  If yes go to Q6 |
| Q5 | If you have no menstrual periods, what is the cause? |  |
| a | Are you pregnant? | Yes/No |
| b | Has your uterus been removed? | Yes/No |
| c | Do you use an intrauterine hormone device? | Yes/No |
| d | Are you in the menopause? | Yes/No |
| e | Do you use estrogen? | Yes/No |
| f. | Other causes? | Yes/No |
|  | **SYMPTOMS FROM THE URINARY TRACT** |  |
| Q6 | How many times do you urinate during the daytime, on average? | _____ |
| Q7 | Do you have to urinate during the night? | Yes  If yes, how many times? ____  No |
| Q8 | Do you have urinary urgency with a sudden and strong urge to void, which is hard to postpone? | Yes  No, if no go to Q10 |
| Q9 | How does your urinary urgency affect you? | No problem  A minor nuisance  Some bother  Much bother  A major problem |
| Q10 | Do you take any medication for urinary urgency? | Yes/No |
| Q11 | Were you a bed wetter during childhood, (involuntary loss of urine whilst sleeping)? | Yes,  If yes at what age did it stop?  No |
| Q12 | Do you have involuntary loss of urine? | Yes/No  If no, go to Q21 |
| Q13 | How often do you have involuntary loss of urine**?** | Less than once a month  Once or more per month  Once or more per week  Every day and/or night |
| Q14 | How much urine do you leak each time? | -a few drops  -small amounts  -large amounts |
| Q15 | Do you have involuntary loss of urine in connection  with coughing, sneezing, laughing, or lifting heavy  items? | Yes/No |
| Q16 | Do you have involuntary loss of urine in connection with a sudden and strong urge to void? | Yes/No |
| Q17 | For how long have you had involuntary loss of  urine? | 0-5 years  5-10 years  More than 10 years |
| Q18 | Have you consulted a doctor because of involuntary  loss of urine? | Yes/No |
| Q19 | How does your urinary leakage affect you? | No problem  A minor nuisance  Some bother  Much bother  A major problem |
| Q20 | If you have given birth- did you have urinary leakage even before the first pregnancy? | Yes/No |
| Q21 | Have you had any surgery for a urinary incontinence? | Yes/No |
| Q22 | Do you take any medication for urinary incontinence? | Yes/No |
| Q23 | Has your mother suffered from urinary leakage? | Yes/No/Do not know |
|  | **SYMPTOMS FROM THE VAGINA** |  |
| Q24 | Do you have a sensation of tissue protrusion (a vaginal bulge) from your vagina? | Often  Sometimes  Infrequently  Never |
| Q25 | Do you suffer from a chafing/a rubbing feeling in your vagina/vulva? | Often  Sometimes  Infrequently  Never |
| Q26 | Do you have to lift the front vaginal wall to start or complete voiding? | Often  Sometimes  Infrequently  Never |
|  | If you have no discomforts from your vagina, proceed to Q29. |  |
| Q27 | Are your symptoms worse during straining, for example, during heavy lifting? | Unchanged  Better  Worse |
| Q28 | How do these vaginal symptoms affect you? | No problem  A minor nuisance  Some bother  Much bother  A major problem |
| Q29 | Have you received (any) treatment for a prolapse? | Yes/No |
| Q30 | Have you had any surgery for a prolapse? | Yes/No |
| Q31 | Has your mother suffered from prolapse? | Yes/No/Do not know |
|  | **SYMPTOMS FROM YOUR BACK PASSAGE** |  |
| Q32 | Do you leak solid faeces involuntarily? | Never  Less than once a month  Several times a month but less than once a week  Once a week or more  Once a day or more |
| Q33 | Do you leak liquid faeces involuntarily? | Never  Less than once a month  Several times a month but less than once a week  Once a week or more  Once a day or more |
| Q34 | Do you leak flatus/gas involuntarily? | Never  Less than once a month  Several times a month but less than once a week  Once a week or more  Once a day or more |
| Q35 | Do you use a protective product/pad because of involuntary leakage from the back passage? | Never  Less than once a month  Several times a month but less than once a week  Once a week or more  Once a day or more |
| Q36 | Is your daily life style affected by involuntary leakage from your back passage? | Never  Less than once a month  Several times a month but less than once a week  Once a week or more  Once a day or more |
| Q37 | How do your bowel symptoms affect you? | No problem  A minor nuisance  Some bother  Much bother  A major problem |
| Q38 | Have you received (any) treatment for leakage of flatus/gas or faeces? | Yes/No |
| Q39 | Has your mother suffered from leakage of flatus/gas or faeces? | Yes/No/Do not know |
| Q40 | On the lines below there is room for your own comments regarding this questionnaire | ………………………………. |
